# Voluntary and Involuntary Singlehood and Young Adults’ Mental Health: an Investigation of Mediating Role of Romantic Loneliness

**DOI:** 10.1007/s12144-016-9478-3

**Published:** 2016-07-19

**Authors:** Katarzyna Adamczyk

**Affiliations:** Institute of Psychology, Adam Mickiewicz University in Poznań, ul. A. Szamarzewskiego 89/AB, 60-568 Poznań, Poland

**Keywords:** Voluntary singlehood, Involuntary singlehood, Positive mental health, Mental health illness, Romantic loneliness, Young adults

## Abstract

The present study tested the hypothesis that single young adults who perceive their singlehood as voluntary would report a higher level of positive mental health (i.e., emotional, psychological and social well-being), lower levels of mental health illness (i.e., somatic symptoms, anxiety, social dysfunction, severe depression) and romantic loneliness in comparison to young adults who perceive their singlehood as involuntary. This paper also investigated whether romantic loneliness mediates the relationship between voluntary and involuntary singlehood, positive mental health, and mental health illness. The study sample included 151 participants (86 females and 65 males) aged 20–26 (*M* = 22.48, *SD* = 2.01) from Poland. The main findings were that voluntarily single young adults reported a lower level of romantic loneliness compared to involuntarily single young adults. The two groups differed neither in regard to positive mental health nor in regard to mental health problems. In addition, gender differences were observed solely in the domain of romantic loneliness, with women reporting greater romantic loneliness than men. The mediation analysis revealed that romantic loneliness does not mediate the relationship between voluntary and involuntary singlehood, positive mental health, and mental health illness. Voluntary and involuntary singlehood was predictive of somatic symptoms, anxiety and insomnia, severe depression, and romantic loneliness.

## Introduction

The breadth and depth of an individual’s social connections is predictive of subjective well-being, and social connections (such as the spouse, close friends and confidants, friendly neighbors, and supportive coworkers) decrease the likelihood of sadness, loneliness, low self-esteem and problems with eating and sleeping (Helliwell and Putnam 2004). Prior studies revealed that regardless of the method of measurement of mental health (i.e., diagnoses, symptoms, overall psychological well-being, psychiatric treatment), married people reported the best health compared to never-married and formerly married people (e.g., Barrett 2000). Moreover, prior research also provided evidence of the linkage not only between mental health and marital status, but also between mental health and non-marital relationships (e.g., Adamczyk and Segrin 2015a; Braithwaite et al. 2010). At the same time, in most Western countries we can observe the diminishing position of marriage in people’s lives (Næss et al. 2015), in particular in young adults’ lives who postpone marriage and prolong their premarital relationships (Lehnart et al. 2010). These changes are accompanied by higher social acceptance of alternatives to marriage, such as non-marital heterosexual cohabitation and singlehood (Glenn and Weaver 1988). As a result, the psychological advantages of marriage over singlehood have been suggested to become weaker (Glenn and Weaver 1988).

While the number of single persons has been on the rise, in particular in the case of those who declare choosing to be single, it is important to investigate whether and how voluntary and involuntary singlehood affects the psychosocial functioning of single young adults. This issue is gaining in importance in light of the fact that although remaining single is becoming prolonged with respect to individuals’ lifespan and is increasingly more prevalent, remaining single – especially by choice – leads to negative perception of people making such choices. For example, Morris and Osburn (2016) found in their study that singles who had chose to remain single were perceived more negatively (as being more self-centered and less well-adjusted) than singles who wanted to marry. The issue of involuntary singlehood is not limited to remaining single and experiencing the unmet need to have a partner/spouse; it also raises the question about other life spheres that might be affected by involuntary singlehood, in particular when singlehood extends over time and continues in young, middle and late adulthood. Involuntary singlehood may, therefore, be related to certain negative effects, for example involuntary childlessness and unmet parenthood goals. In turn, involuntarily childless people experience a number of physiological and psychological symptoms of distress (e.g., health complaints, depression, anxiety and even complicated bereavement) (Lechner et al. 2006).

The present paper focuses on singlehood understood as voluntary (i.e., a result of an individual’s choice) and involuntary (i.e., as related to external factors, thus not experienced by choice). Therefore, in this paper the nature of voluntary or involuntary singlehood is related to the subjective perception of singlehood by an individual in terms of his or her own choice or external barriers hindering finding a partner and/or remaining in a relationship, rather than to more objective circumstances leading to involuntary singlehood such as, for instance, in China, where as a result of the unbalanced sex ratio at birth, excess female child mortality and increasing female marriage migration, the male marriage squeeze led to difficulties among men in some rural areas in finding a wife (Liu et al. 2014). Although the linkage between marital status, romantic relationships and mental health is strongly established, few studies have investigated the linkage between voluntary and involuntary singlehood and mental health. Therefore, the primary aim of this paper is to provide a deeper insight into singlehood from the perspective of its voluntary or involuntary nature. In order to achieve this aim, the present study intended to investigate possible differences in the domain of positive mental health (i.e., emotional, psychological and social well-being), mental health illness (i.e., somatic symptoms, anxiety and insomnia, social dysfunction, and severe depression) and romantic loneliness between voluntarily and involuntarily single individuals from Poland. The study also focused on possible gender differences as prior research suggested that marriage and romantic relationships may operate differently for women and men (Simon 2002; Wadsworth 2016) and that certain gender differences exist in the domain of romantic loneliness and mental health (e.g., Dykstra and de Jong Gierveld 2004; Simon 2002). The second major objective of the paper is to explore a theoretical model postulating the mediating role of romantic loneliness in the linkage between voluntary and involuntary singlehood and young adults’ positive mental health and mental health illness. It is important to emphasize that the sample of Polish young adults examined in the current investigation provides a useful context for a test of these interconnections since most people associate Poland with strong Catholic values and low acceptance of alternative forms of marital and family life (Baranowska-Rataj et al. 2013). Such dominant pro-marriage culture may negatively affect mental health of single people (Adamczyk and Segrin 2015b) as, for example, the association between marital status and subjective well-being may depend on the marital context, i.e., the degree to which marriage is recognized as a normative expectation or achievement by a given peer group (Wadsworth 2016).

## Voluntary and Involuntary Singlehood

In regard to the choice of whether to remain single, undoubtedly some persons chose single life and prefer such a lifestyle (Boyd and Bee 2008; Braun-Gałkowska 2008; Lewis and Moon 1998), but at the same time, for real or imagined reasons, some people do not find a lifetime partner (Lewis and Moon 1998). Prior research attempted to investigate the reasons behind singlehood. For example, Frazier et al. (1996) in their study based on 217 heterosexual divorced and never-married adults aged 31–68 years (*M* = 43) identified the following attributions regarding reasons for being unmarried: (1) not meeting the right person, (2) not meeting potential partners, (3) marriage as not a priority in life, (4) importance of other things in life, (5) choice of being single, (6) difficulties in establishing relationships; (7) fear that the relationship will not work, (8) fear of commitment; (9) belief that all good partners are already “taken.” Out of these reasons, the choice of being single was the fifth most frequently provided explanation. In the same study, when asked in an open-ended question about their reasons for being single, the respondents also listed the choice of being single (the second most frequently indicated category). In a study by Gigy (1980) in which 66 single women (of 30 years of age or more) took part, the choice of being single was also indicated as one of the reasons for being unmarried. In a study performed on a sample of 160 women (of 30 and 60 years of age and more) from Jammu and Delhi, Prabhakar (2011) found that the two main reasons for remaining single were the individual’s voluntary decision and circumstantial factors. The first category included reasons such as high marital expectations, desire for independence, pursuit of career, disappointment in love, and parental objection to choice marriage, while the second category included financial constraints, loss of parents, inability to find a suitable mate in one’s own caste, and health /disability (Prabhakar 2011).

In general, prior research revealed the following three primary reasons for being unmarried reported by single adults: (1) personal choice, (2) external circumstances, and (3) personal deficits or self-blame (e.g., Austrom and Hanel 1985; Frazier et al. 1996. The first category refers to having positive reasons for being single (e.g., “present lifestyle could not be improved by marriage” or “the lack of need to involve in a relationship”; Austrom and Hanel 1985; Palus 2010). The second category includes single adults indicating external circumstances or “barriers” as reasons for their singlehood (e.g., “not meeting the right person” or “unreciprocated feelings”; Frazier et al. 1996; Palus 2010). In turn, the third category pertains to personal deficits such as shyness or sense of being unattractive (Austrom and Hanel 1985; Palus 2010). Moreover, based on his two primary dimensions to the experience of singlehood (a choice and a temporal dimension), Stein (1981) proposed the following four types of single adults: voluntary temporary, voluntary stable, involuntary temporary, and involuntary stable. At the same time, Stein (1981) and other researchers (e.g., Reynolds et al. 2007) recognized that this classification is a flexible process rather than a stable categorization. Moreover, as Reynolds et al. (2007) indicated, the perception of one’ own singlehood as made by choice or as made by chance may be associated with different outcomes. For instance, individuals who represent themselves as having made a choice to be single and for whom having an intimate relationship is not a central goal in life may not feel that they have failed to achieve this goal. In turn, individuals who want to be committed in a serious relationship, may have to deal with the sense of failure in achieving this goal and they may attribute themselves less agency than those who chose to remain single (Reynolds et al. 2007). This different perception of one’s own singlehood may reflect more general concepts of autonomy and self-determination (e.g., Deci and Ryan 2008). Moreover, control over self and over the environment is related to a wide spectrum of positive outcomes in various life domains, for example satisfaction, physical and psychological well-being (Hostetler 2009). In addition, prior studies, however concerning involuntary celibacy, showed that involuntary celibacy was associated with feelings of sexual frustration, depression, rejection, problems with concentration or work, and low self-esteem (Donnelly and Burgess 2008). Therefore, it is plausible to assume that individuals who perceive their singlehood as chosen may experience greater freedom in making their own choices and taking actions regarding their single life than individuals who perceive their singlehood as being beyond their control. As a result, chosen singlehood might be accompanied by greater positive mental health and lower levels of mental health problems and romantic loneliness.

## Mental Health

Recently the concept of mental health has extended beyond the simple definition of the absence of psychopathologies such as depression and anxiety (see Keyes 2002; Lamers et al. 2011; Westerhof and Keyes 2010). Alongside the assessment of mental health in terms of internalizing symptoms (such as depression and anxiety) and externalizing symptoms (such as alcohol and substance abuse), mental health is also conceptualized as well-being that is related to subjective well-being, psychological well-being, and life satisfaction (Bierman et al. 2006). Mental health is, therefore, understood as “a positive phenomenon that is more than the absence of mental illness” (Westerhof and Keyes 2010, p. 110), as “a syndrome of symptoms of positive feelings and positive functioning in life” (Keyes 2002, p. 208). One of the operationalizations of mental health is subjective well-being, which has been investigated within the following two research traditions (Keyes and Simoes 2012): (1) *hedonic* tradition, in line with which well-being involves happiness and pleasant emotions; and maximizing positive, pleasant feelings, and minimizing negative, unpleasant feelings contributes to the increase of mental health (Lamers et al. 2011). This aspect of the hedonic tradition has been widely investigated in studies on emotional well-being, in which measures of satisfaction with life and positive affect are used (Keyes and Simoes 2012; Lamers et al. 2011; Westerhof and Keyes 2010); (2) *eudaimonic* tradition, which focuses on optimal psychological functioning in life and is referred to as psychological well-being (i.e., the subjective evaluation of optimal individual functioning) and social well-being (i.e., the subjective evaluation of optimal functioning for a community (Lamers et al. 2011; Westerhof and Keyes 2010). In regard to Keyes’ (2002) model of mental health, only a combination of emotional, psychological and social well-being allows for the consideration of mental health.

On a general level, the feeling of being connected with other people can lower morbidity and mortality, and the quality of relationships is a predictor of physical and psychological outcomes in the domain of health (Gore 2014). In their study, Kamp Dush and Amato (2005) concluded – in accordance with prior studies – that romantic relationships (marriage, cohabitation, steady dating) provide benefits for individuals’ mental health and sense of well-being. In line with this notion, in prior research single individuals when compared with married individuals reported higher levels of depression, anxiety, mood disorders, adjustment problems, and other forms of psychological distress, and a higher rate of alcohol-related problems (see Braithwaite et al. 2010). When compared with individuals in non-marital relationship, single individuals also reported more mental health problems (Braithwaite et al. 2010) and lower emotional well-being (Adamczyk and Segrin 2015a). Single people were found to have the lowest level of well-being, followed by dating, cohabiting, and married young adults, who reported the highest levels (Soons and Liefbroer 2008). At the same time, contrary to prior research, in a recent study (Adamczyk and Segrin 2015a) single young adults did not differ in regard to social and psychological well-being and total well-being, as well as in regard to somatic symptoms, anxiety and insomnia, social dysfunction, severe depression, and total mental health illness when compared to their counterparts in non-marital relationships.

With respect to gender differences in the domain of mental health, numerous studies provided the following results (see Simon 2002 for review): (1) married women and men experience better mental health than unmarried women and men; (2) regardless of their marital status, women report more mental health problems than men; however, these studies focused on women’s typical emotional problems; (3) there is an interaction between gender and marital status, with inconsistent findings showing men to derive more emotional benefits from marriage or women deriving benefits from marriage. Simon (2002), in his study ran on a US sample, found that for both women and men marriage and lack of marriage are related to emotional benefits and emotional costs (with the exception of separation and divorce). In his study he found that in the case of all marital statuses women reported more depression, while men reported more substance abuse (Simon 2002). In a study using data from the British Household Panel Survey, men in first partnerships reported better mental health than those who remained single, while single women experienced equally good mental health as did women in their first partnership and better health than those who had experienced a partnership split (Willitts et al. 2004). Therefore, the author emphasizes that gender differences in adults’ mental health should be explained in reference to the function of emotional-socialization experiences. In a study by Bierman et al. (2006), a small number of marginally significant differences were found between men and women with respect to the mental health advantage of the married. In their study, Simon and Barrett (2010) found that relationship status was more important for young women’s than for young men’s emotional well-being. In addition, a break-up of a recent romantic relationship was related to more depression for women than for men, and a current romantic involvement was related to fewer substance abuse problems for women (Simon and Barrett 2010).

## Romantic Loneliness

Loneliness is considered to be a common life experience viewed as a subjectively unpleasant and distressing feeling, and is recognized to be a risk factor for various physiological and health outcomes (Cacioppo et al. 2006). Specifically, regardless of objective social isolation or social support, loneliness has been found to be related to negative outcomes in the domain of physical health (e.g., poorer immune functioning, poorer cardiovascular functioning, impaired sleep, obesity) and to personality disorders, hypochondriasis, schizophrenia, suicidal ideation and behavior, depression, and anxiety (Aanes et al. 2010; Cacioppo et al. 2006). Aanes et al. (2010) in their study found that the importance of loneliness as a mediator of the linkage between interpersonal stress and health outcomes (i.e., anxiety symptoms, depressive symptoms, and somatic symptoms) differs for these outcomes. To be precise, the authors found that in the case of depressive symptoms over 75 % of the total effect was mediated through loneliness, whereas in the case of somatic symptoms just over 40 % of the total effect was mediated through loneliness. In a more recent study, individuals with the worst mental health and well-being were three to five times more likely to report occasional loneliness and three to six times more likely to report frequent loneliness (Kearns et al. 2015). Furthermore, in a Russian study, lonely individuals were characterized by a significantly increased risk of reporting poor self-rated health, mental health problems and insomnia in the previous twelve months (Stickley et al. 2015).

Loneliness may be conceptualized as a multifaceted and domain-specific phenomenon. Weiss (1973) was the first to describe loneliness as a multidimensional experience and proposed a distinction between social loneliness as a result of an inadequate access to a network of peers, co-workers, neighbours, or friends, and emotional loneliness resulting from a lack of close or intimate relationships that are characteristic of ties with a romantic partner, parent, or child. Emotional loneliness is primarily related to “the absence of a partner, that is, with the absence of an exclusive, close, and intimate tie” (Dykstra and Fokkema 2007, p. 9). In turn, social loneliness is related to a perceived deficiency in social networks, or a lack of social relations or social activities (Russell et al. 1984; Weiss 1973). Furthermore, on the basis of Weiss’ (1973) distinction between the experience of social isolation (social loneliness) and emotional isolation (emotional loneliness), DiTommaso and Spinner (1993) noted that emotional loneliness appeared to be comprised of two domains, that is, family emotional loneliness and romantic emotional loneliness. The lack of romantic partners or intimate relationships may be an important perceived causal factor for one’s present feelings of loneliness (e.g., Rokach and Brock 1998). For example, married individuals and individuals living with a significant other reported less romantic loneliness than those who were not in such relationships (Bernardon et al. 2011). DiTommaso and Spinner (1993) revealed that being involved in a romantic relationship was significantly related to lower levels of romantic loneliness, but was only weakly linked to family and social loneliness. Divorce or widowhood were found to be associated with an increased risk of feeling lonely, whereas not living alone and having more social support turned out to lower the risk of being lonely (Stickley et al. 2015). Furthermore, several studies conducted in Poland also provided consistent results demonstrating that single young adults report greater romantic loneliness than young adults in non-marital relationships (e.g., Adamczyk 2015).

In regard to gender differences in the domain of loneliness, the results of past studies are not congruent. In other words, some prior studies revealed that men experienced greater loneliness than women (e.g., Dykstra and de Jong Gierveld 2004), whereas other studies indicated no differences (Cramer and Neyedley 1998) or women reporting greater loneliness (e.g., Jakobsson and Hallberg 2005). In other studies, male university students had higher levels of romantic loneliness, while there were no significant gender differences for either social or family loneliness (DiTommaso et al. 2003). Furthermore, in a study by DiTommaso et al. (2005), men reported higher levels of family and social loneliness than did women. In turn, DiTommaso et al. (2007), in a study utilizing a sample of individuals aged 17 to 79 years, did not find significant gender differences in the area of three distinct domains of loneliness. In a recent Polish study men were found to experience greater social loneliness than women, but no gender differences emerged in the domain of romantic and family loneliness (Adamczyk 2015).

## An Exploration of Mediation Model

**Fig. 1:**
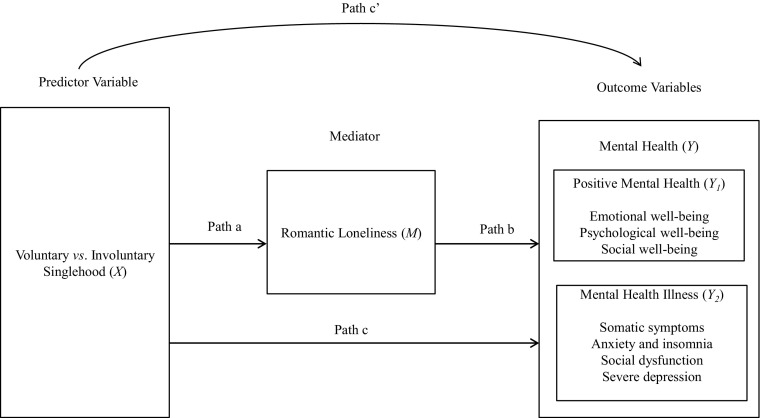
Conceptual Model of Hypothesized Mediation: Voluntary vs. Involuntary as Predictor, and Romantic Loneliness as Mediator of Positive Mental Health and Mental Health Illness

As discussed in the previous section, there is a well-established linkage between marital status (and non-marital relationships) and mental health, as well as between loneliness and mental health outcomes. At the same time, to the best of my knowledge, there is little research investigating these associations in reference to voluntary and involuntary singlehood and a specific type of emotional loneliness, that is, romantic loneliness. Therefore, the current study also intended to explore the theoretical model in which romantic loneliness is postulated to operate as a mediator of the linkage between voluntary and involuntary singlehood, positive mental health (i.e., emotional, psychological and social well-being) and mental health illness (i.e., somatic symptoms, anxiety and insomnia, social dysfunction, and severe depression) (see Fig. 1). In addition, since prior research suggested possible gender differences in the domain of romantic loneliness and mental health, the hypothesized mediation model was also intended to be tested separately in a sample of women and men.

As shown in Fig. 1, there are four paths in the model to be investigated: (1) *Path c* pertaining to the relation between the predictor (voluntary and involuntary singlehood) and the outcomes (positive mental health and mental health illness). Considering that prior research attempting to empirically link these constructs is scarce, it is, however, plausible to assume that voluntary singlehood will be related to more positive outcomes (a higher level of positive mental health and a lower level of mental health illness) than involuntary singlehood; (2) *Path a* pertaining to the relation between the predictor (voluntary and involuntary singlehood) and the mediator (romantic loneliness). Similarly, although no prior study examined the linkage between voluntary and involuntary singlehood and romantic loneliness, it is plausible to expect that voluntary singlehood will be related to lower romantic loneliness; (3) *Path b* pertaining to the relation between the mediator (romantic loneliness) and the outcome variables (positive mental health and mental health illness). The studies cited in previous sections provide strong evidence for the linkage between loneliness and mental health outcomes. Therefore, it is possible that also romantic loneliness will be associated with mental health outcomes, in particular higher levels of loneliness will be related to lower positive mental health and greater mental health illness; (4) *Path c’* pertaining to the relation between the predictor (voluntary and involuntary singlehood) and the outcomes (positive mental health and mental health illness) when the mediator (romantic loneliness) is included in the model. This path was intended to be examined in the current study.

## Present Study

### Research Objectives and Hypotheses

The aim of the current study was twofold. The first objective was to investigate the possible differences between voluntarily and involuntarily single young adults, as well as between women and men in the domain of mental health (positive mental health and mental health illness) and romantic loneliness. Based on the literature presented in the previous sections, it was expected that:

- Hypothesis 1: Voluntarily single young adults will report a higher level of positive mental health (i.e., emotional, psychological and social well-being) and total well-being than involuntarily single young adults.
- Hypothesis 2. Voluntarily single young adults will report a lower level of mental health illness (i.e., somatic symptoms, anxiety and insomnia, social dysfunction, and severe depression) and total mental health illness than involuntarily single young adults.
- Hypothesis 3. Voluntarily single young adults will report lower level of romantic loneliness than involuntarily single young adults.

With respect to gender differences elaborated in the theoretical part of the paper, and considering that prior studies provided inconsistent results with respect to gender differences, two open research questions were formulated:

RQ1. Will women and men report similar levels of positive mental health, mental health illness, and romantic loneliness?

RQ2. Will be there an interaction of voluntary and involuntary singlehood and gender in the domain of positive mental health, mental health illness, and romantic loneliness?

The second major aim of the study was to explore the theoretical model in which romantic loneliness was postulated to be a mediator of the linkage between voluntary and involuntary singlehood and young adults’ positive mental health and mental health illness. Therefore, the following hypothesis was formulated:

- Hypothesis 4: Romantic loneliness will mediate the association between voluntary and involuntary singlehood and young adults’ positive mental health and mental health illness.

## Method

### Participants and Procedure

The study was carried out on a sample of university students from different faculties at a Polish university and non-students. Five hundred questionnaires were originally distributed. A total of 320 students and non-students returned questionnaires (64 % response rate).

Of these, 169 participants were removed because they were involved in a non-marital romantic relationships, married, divorced, separated, single without declaration if singlehood is to be perceived as voluntary or involuntary, or due to incomplete data, yielding a final sample of 151 single, heterosexual, never married, childless participants. University students constituted 68 % of the total sample (*n* = 103), while non-student participants with higher education level constituted 32 % of the total sample (*n* = 48). The age of participants ranged from 20 to 26 years, with the average being 22.48, and standard deviation of 2.01. Participants resided in a large Polish city with a population exceeding 500,000 inhabitants. Women represented 57 % (*n* = 86) and men 43 % of the sample (*n* = 65). Thirty respondents (19.87 %) indicated that in the past they had sought psychological/psychiatric help, whereas 121 respondents (80.13 %) indicated that in the past they had not sought this type of help.

All participants fitted into one of the two types of singlehood: (1) voluntary singlehood or (2) involuntary singlehood. First, being single was defined as “not in a committed relationship for at least 6 or more months, but wanting to become committed in the near future (within the next year or so)”, and being in a non-marital romantic relationship was defined as “in a committed non-marital relationship for at least 6 or more months, and wanting to be committed in the near future (within the next year or so)” (see Schachner et al. 2008). The criterion of 6 months was used to distinguish between single and partnered individuals arbitrary. It was based on prior study performed by Donnelly and Burgess (2008), which, however, referred to involuntary celibacy within long-term partnered relationship not to the lack of a lifetime partner. This criterion, however, helped to include people about whom we may say that their singlehood is a rather long-term situation rather than a short-term situation. Regarding this criterion, all participants who were single for a period shorter than 6 months were excluded from further analysis. Second, in the current study, voluntary singlehood was defined as being single for at least 6 months by one’s own decision, whereas involuntary singlehood was defined as being single for at least 6 months due to external circumstances perceived by an individual as not depending on him or her (see Donnelly and Burgess 2008). Participants who perceived their singlehood as voluntary constituted 53.60 % (*n* = 81) of the analyzed sample, whereas participants who perceived their singlehood as involuntary represented the remaining 46.40 % (*n* = 70) of the sample. Thirty three persons declared that they had never had a partner. The average duration of remaining single was 5.08 years, standard deviation of 7.35 years among 118 respondents who provided the duration of their singlehood. One hundred and one participants declared that they would like to have a partner in the future, while 10 participants declared that they would not like to have a partner in future. In terms of ethnicity, the sample included 100 % of Poles. In terms of religion, 106 participants (70.20 %) reported to be Catholic, whereas 45 participants (29.80 %) declared to be atheist.

The sample was recruited by author by distribution of the questionnaires through university students who were also asked to refer members of their social networks to participate in the investigation. The questionnaire packages were administered in classrooms to groups of 20 to 30 students at a time and participation was voluntary. The nonstudent participants were obtained through university students who passed questionnaires to members of their social networks. At the same time, university students were specifically instructed to not recruit their romantic partners and relatives into the study, but they were allowed to recruit friends. The purpose of the study was explained to participants along with an assurance of anonymity and explanation of their freedom to withdraw from the study without consequence. The study was conducted according to the ethical guidelines in the Polish Code of Professional Ethics for the Psychologist that apply to psychologists who are researchers and practitioners. Participants were not offered any compensation for their participation in the study.

### Measures

#### Demographic Variables

The demographic variables in the study were as follows: age, gender, place of residence, education level, possessing children, sexual orientation, religion, current relationship status, duration of being single or being in a relationship, and desire to possess a partner in the future. These variables were assessed with straightforward single-item questions.

#### Mental Health - Emotional, Psychological and Social well-Being

To measure emotional and psychological well-being the Mental Health Continuum - Short Form (MHC – SF; Keyes 2009) (Polish adaptation – Karaś et al. 2014) was used. The MHC-SF consists of 14 items measuring emotional, psychological and social well-being. In the current study emotional and psychological well-being were used. Respondents are asked to answer questions about how they have been feeling during the past month using a scale ranging from *0* (never) to *5* (every day). Example items are: “During the past month, how often did you feel happy?” (emotional well-being) and “During the past month, how often did you feel that you had warm and trusting relationships with others?” (psychological well-being). The short form of the MHC has shown excellent internal consistency (> .80) and discriminant validity in the case of adolescents (ages 12–18) and adults in the U.S., the Netherlands and South Africa (Keyes 2009). In the present study the internal consistency for the subscales was as follows: α = .89 for Emotional well-being, *α* = .84 for Psychological well-being, α = .75 for Social well-being, and α = .90 for the Total mental health.

#### Mental Health Illness

To measure mental health illness the General Health Questionnaire***-***28 (GHQ-28; Goldberg and Hillier 1979) (Polish adaptation – Goldberg et al. 2001). The General Health Questionnaire is a self-administered questionnaire used to measure non-psychotic psychiatric disorders (Goldberg and Hillier 1979). The GHQ-28 scale was derived from the original 60-item version of the questionnaire mainly for research purposes but it is also often used as a measure of psychological well-being (e.g., Goldberg and Williams 1988). GHQ-28 consists of four 7-item scales: somatic symptoms, anxiety and insomnia, social dysfunction, and severe depression. The respondent is asked to compare his recent psychological state with his usual state on a 4-point response scale. For each item the four possible answers are as follows:1 – not at all, 2 – no more than usual, 3 – rather more than usual, 4 – much more than usual. In the current study the bimodal scoring procedure (0, 0, 1, 1) was applied. Using the conventional bimodal GHQ scoring method there is a range of 0–28 with a score above a threshold of 4 indicative of psychiatric disorder. In the present study the internal consistency for the subscales was the following: α = .73 for Somatic symptoms, α = .81 for Anxiety, α = .75 for Social dysfunction, α = .82 for Severe depression, and α = .90 for the Total scale.

#### Romantic Loneliness

I used the 5-item romantic loneliness subscale from The Social and Emotional Loneliness Scale for Adults - Short Form (SELSA-S; DiTommaso et al. 2004) (Polish adaptation - Adamczyk and DiTommaso 2014), using 7-point Likert-type scale, ranging from *1* (strongly disagree) to *7* (strongly agree), to measure romantic loneliness. This subscale has demonstrated adequate validity and reliability in prior research (e.g., DiTommaso et al. 2004). An example item is “In the last month had a romantic partner with whom I shared my most intimate thoughts and feelings.” In the present study, Cronbach’s alpha coefficient for romantic loneliness subscale was α = .70.

#### Voluntary Vs. Involuntary Singlehood

The voluntary vs. involuntary singlehood was assessed with the following item: “Being single is a result of …” (options “My decision” or “External circumstances beyond my control”).

## Results

### Preliminary Analyses

As a starting point, a univariate analysis of variance was performed on the demographic variables to evaluate the mean differences in the variables between voluntary and involuntary single young adults as well as between women and men.

Results indicated that voluntarily and involuntarily single individuals did not differ in regard to their age, *F*(1, 147) = 0.90, *p* = 348, *η* ^*2*^ = .01, gender, χ^2^(1, *N* = 151) = 1.86, *p* = .173, place of residence, Cramer’s V (5, *N* = 151) = .23, *p* = .156, education level, Cramer’s V (4, *N* = 151) = .14, *p* = .562, or the duration of remaining single, *F*(1114) = 3.33, *p* = .071, *η* ^*2*^ = .03. Both groups also did not differ in regard to their use of psychological/psychiatric help in the past, χ^2^(1, *N* = 151) = 0.50, *p* = .494.

With respect to gender, women and men differed in regard to age with men being older (*M* = 23.08*, SD* = 2.62) than women (*M* = 22.29, *SD* = 2.10), *F*(1) = 5.33, *p* = .022, *η* ^*2*^ = .04. Significant differences were observed between women and men in regard to their education level, Cramer’s V (4, *N* = 151) = .27, *p* = .030, with a greater number of women reporting higher education levels than men. Women and men did not differ in regard to their duration of remaining single, *F*(1, 114) = 1.88, =.174, *η* ^*2*^ = .02, but at the same time there was an interaction of voluntary vs. involuntary singlehood and gender for the duration of remaining single, *F*(1, 114) = 6.02, *p* = .016, *η* ^*2*^ = .05. In an attempt to explain this interactional effect, an analysis of the simple main effect of voluntary vs. involuntary singlehood in the group of women and men was performed. Results of this analysis indicated a significant simple main effect of voluntary and involuntary singlehood and gender for the duration of remaining single in the group of men, *F*(1, 49) = 4.54, *p* = .038, *η* ^*2*^ = .09, whereas a simple main effect of voluntary and involuntary singlehood and gender for the duration of remaining single in the group of women occurred to be nonsignificant, *F*(1, 65) = 0.54, *p* = .466, *η* ^*2*^ = .01. Voluntarily single men reported lower duration of remaining single (*M* = 17.67 months, *SD* = 18.23 months) than involuntarily single men (*M* = 39.28 months, *SD* = 53.14 months). Women and men did not differ in regard to their place of residence, Cramer’s V (5, *N* = 151) = .20, *p* = .313, and use of psychological/psychiatric help in the past, χ^2^(1, *N* = 151) = 2.83, *p* = .092.

Next, the bivariate correlations among the major variables were assessed (see Table 1).

**Table 1:** Bevariate correlations among major variables

Variables	1	2	3	4	5	6	7	8	9	10
1. Emotional well-being	-	.70***	.58***	.84***	−.23**	−.41***	−.48***	−.45***	−.49***	−.01
2. Psychological well-being		-	.62***	.92***	−.23***	−.39***	−.41***	−.34***	−.46***	−.14
3. Social well-being			-	.85***	−.12	−.27**	−.21**	−.34***	−.29***	−.12
4. Total well-being				-	−.22**	−.40***	−.41***	−.47***	−.47***	−.12
5. Somatic symptoms					-	.54***	.39***	.30***	.74***	.20*
6. Anxiety and insomnia						-	.55***	.53***	.86***	.12
7. Social dysfunction							-	.56***	.78***	.07
8. Severe depression								-	.74	.07
9. Total mental health illness									-	.15
10. Romantic loneliness										-

*N* = 151

*** *p* < .001; ** *p* < .01; * *p* < .05

Cohen’s (1988) benchmarks were used and correlations of .20 as small, correlations of .30 as moderate, and correlations of .50 as large were regarded. Results indicated that most of the correlations were moderate and strong. Only the correlations between measurements of well-being and romantic loneliness were insignificant, with the exception of the correlation between somatic symptoms and romantic loneliness, which was moderate and positive.

### Substantive Analyses

#### Differences in Positive Mental Health

Regarding the strong correlations between emotional, psychological and social well-being, (see Table 2) a multivariate analysis of variance (MANOVA) was performed to examine the differences between voluntarily and involuntarily single young adults.

**Table 2:** Means, standard deviations, effect sizes, and significance levels for voluntarily and involuntarily single individuals

	Total sample (*N* = 151)	Voluntarily single individuals (*n* = 81)	Involuntarily single individuals (*n* = 70)	*F value*	*η2*
Variables	Mean (SD)	Mean (SD)	Mean (SD)		
Multivariate test				1.62	.03
*Mental health*					
Emotional well-being	9.42 (3.47)	9.63 (3.47)	9.19 (3.49)	0.36	.00
Psychological well-being	16.85 (6.41)	17.81 (6.37)	15.74 (6.32)	3.47	.02
Social well-being	10.67 (5.11)	10.86 (5.27)	10.44 (4.95)	0.19	.00
Total well-being	36.95 (13.10)	38.31 (13.28)	35.37 (12.80)	1.52	.01
Multivariate test				2.03	.05
*Mental health illness*					
Somatic symptoms	1.87 (1.93)	1.56 (1.88)	2.23 (1.93)	3.64	.03
Anxiety and insomnia	1.74 (2.03)	1.31 (1.70)	2.23 (2.27)	7.29**	.05
Social dysfunction	1.08 (1.58)	0.83 (1.39)	1.35 (1.73)	3.02	.02
Severe depression	0.83 (1.57)	0.57 (1.31)	1.13 (1.78)	4.20*	.03
Total mental health illness	5.50 (5.55)	4.26 (4.71)	6.94 (6.11)	7.63**	.05
Univariate test					
Romantic loneliness	21.44 (6.22)	18.95 (6.29)	24.32 (4.76)	32.56***	.18

*** *p* < .001; ** *p* < .01; * *p* < .05

The performed analysis revealed a nonsignificant multivariate effect of voluntary vs. involuntary singlehood on emotional, psychological, social and total well-being, Wilks’s Λ = .97, *F*(3, 145) = 1.62, *p* = .147, *η* ^*2*^ = .03. Voluntarily and involuntarily single young adults reported similar levels of emotional well-being, *F*(1, 147) = 0.36, *p* = .555, *η* ^*2*^ = .00, psychological well-being, *F*(1, 147) = 3.47, *p* = .064, *η* ^*2*^ = .02, social well-being, *F*(1, 147) = 0.19, *p* = .666, *η* ^*2*^ = .00, and total well-being, *F*(1, 147) = 1.52, *p* = .219, *η* ^*2*^ = .01.

#### Differences in Mental Health Illness

As with positive mental health, regarding the strong correlations between indicators of mental health illness (see Table 2), a multivariate analysis of variance (MANOVA) was performed to examine the differences between voluntarily and involuntarily single young adults.

The performed analysis revealed a nonsignificant multivariate effect of voluntary vs. involuntary singlehood on somatic symptoms, anxiety and insomnia, social dysfunction, severe depression, and total mental health illness, Wilks’s Λ = .95, *F*(4, 144) = 2.03, *p* = .094, *η* ^*2*^ = .05.

As Table 2 shows, voluntarily and involuntarily single young adults did not differ in regard to somatic symptoms, *F*(1, 147) = 3.64, *p* = .058, *η* ^*2*^ = .02, anxiety and insomnia, *F*(1, 147) = 7.29, *p* = .008, *η* ^*2*^ = .05, social dysfunction, *F*(1, 147) = 3.02, *p* = .085, *η* ^*2*^ = .02, severe depression, *F*(1, 147) = 4.20, *p* = .042, *η* ^*2*^ = .03, and total mental health illness, *F*(1, 147) = 7.63, *p* = .006, *η* ^*2*^ = .05.

#### Differences in Romantic Loneliness

A univariate analysis of variance (see Table 2) demonstrated that voluntarily single young adults reported lower romantic loneliness than involuntarily single young adults, *F*(1, 147) = 32.56, *p* = .000, *η* ^*2*^ = .18.

#### Gender Differences

With respect to positive mental health, a multivariate analysis of variance (MANOVA) revealed a nonsignificant multivariate effect of gender on positive mental health, Wilks’s Λ = .98, *F*(3, 145) = 0.97, *p* = .410, *η* ^*2*^ = .02. In light of the results of the analysis (see Table 3), it can be stated that no gender differences exist in regard to emotional well-being, *F*(1, 147) = 1.11, *p* = .293, *η* ^*2*^ = .01, psychological well-being, *F*(1, 147) = 1.14, *p* = .288, *η* ^*2*^ = .01,social well-being, *F*(1, 147) = 0.04, *p* = .837, *η* ^*2*^ = .00, and total well-being, *F*(1, 147) = 0.52, *p* = .475, *η* ^*2*^ = .00. The performed analysis also did not reveal an interactional effect of gender and voluntary vs. involuntary singlehood on positive mental health, Wilks’s Λ = .99, *F*(3, 145) = 0.28, *p* = .839, *η* ^*2*^ = .00.

**Table 3:** Means, standard deviations, effect sizes, and significance levels for women and men

	Total sample (*N* = 151)	Women (*n* = 86)	Men (*n* = 65)	*F value*	*η2*
Variables	Mean (SD)	Mean (SD)	Mean (SD)		
Multivariate test				0.97	.02
*Mental health*					
Emotional well-being	9.42 (3.47)	9.15 (3.44)	9.78 (3.52)	1.11	.01
Psychological well-being	16.85 (6.41)	16.27 (6.32)	17.63 (6.51)	1.14	.01
Social well-being	10.67 (5.11)	10.74 (4.88)	10.57 (5.44)	0.04	.00
Total well-being	36.95 (13.10)	36.16 (12.91)	37.98 (13.38)	0.51	.00
Multivariate test					
*Mental health illness*				1.37	.04
Somatic symptoms	1.87(1.93)	2.13 (1.99)	1.52 (1.80)	2.95	.02
Anxiety and insomnia	1.74 (2.03)	1.90 (2.10)	1.52 (1.93)	0.66	.00
Social dysfunction	1.08 (1.58)	1.30 (1.81)	0.77 (1.14)	3.78	.03
Severe depression	0.83 (1.57)	0.95 (1.61)	0.66 (1.51)	0.83	.01
Total mental health illness	5.50 (5.55)	6.28 (5.99)	4.48 (4.76)	2.99	.02
Univariate test					
Romantic loneliness	21.44 (6.22)	22.73 (5.79975	19.73 (6,39,144	6.52	.04

In regard to mental health illness, a multivariate analysis of variance (MANOVA) (see Table 3) revealed a nonsignificant multivariate effect of gender, Wilks’s Λ = .96, *F*(4, 144) = 1.37, *p* = .247, *η* ^*2*^ = .04. Follow-up analyses demonstrated that women and men did not differ in the domain of somatic symptoms, *F*(1, 147) = 2.95, *p* = .088, *η* ^*2*^ = .02, anxiety and insomnia, *F*(1, 147) = 0.66, *p* = .416, *η* ^*2*^ = .00, social dysfunction, *F*(1, 147) = 3.78, *p* = .054, *η* ^*2*^ = .03, severe depression, *F*(1, 147) = 0.83, *p* = .364, *η* ^*2*^ = .00, and total mental health illness, *F*(1, 147) = 2.99, *p* = .086, *η* ^*2*^ = .02. At the same time, no interactional effect of gender and voluntary vs. involuntary singlehood on mental health illness emerged to be significant, Wilks’s Λ = .99, *F*(4, 144) = 0.23, *p* = .922, *η* ^*2*^ = .01.

In the domain of romantic loneliness, a univariate analysis of variance revealed that women reported higher levels of romantic loneliness than men, *F*(1, 147) = 6.52, *p* = .012, *η* ^*2*^ = .04 (see Table 3). At the same time, no interactional effect of gender and voluntary vs. involuntary singlehood was observed to be significant, *F*(1, 147) = 0.86, *p* = .355, *η* ^*2*^ = .01.

### Tests of Mediation Model

The final set of analyses examined the mediating role of romantic loneliness in the linkage between voluntary and involuntary singlehood, positive mental health, and mental health illness (see Figure 1). In order to establish the mediation effect of romantic loneliness, four steps (involving three regression equations) were performed in line with the method for testing mediation in psychological research as outlined by Baron and Kenny’s (1986) and other researchers (Frazier et al. 2004). In order to make them more concise and available in one place, all the results of the testing mediation are presented in Table 4. The tests of mediation effect of romantic loneliness were preformed separately for each of the seven outcomes, that is, for emotional well-being (Outcome 1), psychological well-being (Outcome 2), social well-being (Outcome 3), somatic symptoms (Outcome 4), anxiety and depression (Outcome 5), social dysfunction (Outcome 6), and severe depression (Outcome 7).

**Table 4:** Testing mediator effects using multiple regression

Testing steps in mediation model	*B*	*SE B*	95 % CI	Stand.	*R* ^*2*^
Testing Step 1 (Path c)					
Outcomes (EWB, PWB, SWB, SS, AI, SDYS, SDEP)					
Predictor: VIS^a^ and Outcome: EWB	−0.36	0.57	−1.48, 0.77	−.05	.00
Predictor: VIS and Outcome: PWB	−1.81	1.04	−3.87, 0.25	−.14	.02
Predictor: VIS and Outcome: SWB	−0.47	0.84	−2.13, 1.19	−.05	.00
Predictor: VIS and Outcome: SS	0.63	0.32	0.00, 1.25	.16*	.03
Predictor: VIS and Outcome: AI	0.87	0.33	0.23, 1.51	.21**	.05
Predictor: VIS and Outcome: SDYS	0.44	0.26	−0.08, 0.95	.14	.02
Predictor: VIS and Outcome: SDEP	0.55	0.25	0.05, 1.05	.18*	.03
Testing Step 2 (Path a)					
Outcome: RL					
Predictor: VIS	5.37	0.92	3.56, 7.12	.43***	.18***
Testing Step 3 (Paths b and c’ )					
Outcomes (EWB, PWB, SWB, SS, AI, SDYS, SDEP)					
Mediator: RL (Path b) and Outcome: EWB	−0.01	0.05	−0.11, 0.10	−.01	.00
Predictor: VIS	−0.39	0.63	−1.64, 0.86	−.06	.00
Mediator: RL (Path b) and Outcome: PWB	0.10	0.09	−0.08, 0.29	.10	.03
Predictor: VIS	−1.27	1.15	−3.56, 1.01	−.10	.03
Mediator: RL (Path b) and Outcome: SWB	0.10	0.08	−0.05, 0.25	.12	.00
Predictor: VIS	0.06	0.93	−1.77, 1.89	.01	.00
Mediator: RL (Path b) and Outcome: SS	−0.05	0.03	−0.11, 0.00	−.17	.04
Predictor: VIS	0.34	0.35	−0.34, 1.02	.09	.04
Mediator: RL (Path b) and outcome: AI	−0.01	0.03	−0.07, 0.05	−.04	.03
Predictor: VIS	0.81	0.36	0.10, 1.52	.20*	.03
Mediator: RL (Path b) and SDYS	−0.00	0.02	−0.05, 0.04	−.01	.00
Predictor: VIS	0.42	0.29	−0.15, 0.99	.13	.00
Mediator: RL (Path b) and SDEP	−0.00	0.02	−0.05, 0.04	−.00	.02
Predictor: VIS	0.54	0.28	−0.01, 1.10	.17	.02

*VIS* Voluntary and involuntary singlehood; EWB = Emotional well-being; *PWB* Psychological well-being; *SWB* Social well-being; *SS* Somatic symptoms; *AI* Anxiety and insomnia; *SDYS* Social dysfunction; *SDEP*Severe depression; *RL* Romantic loneliness

^a^0 = voluntary singlehood, 1 = involuntary singlehood

*** *p* < .001; **p < .01; * *p* < .05

In the first step, the outcome variables (see the above-mentioned outcomes) were regressed on the predictor (voluntary and involuntary singlehood) to establish if there is an effect to mediate (see Path c in Fig. 1). The simple linear regression analysis revealed that Path c was significant only for somatic symptoms, anxiety and insomnia, and severe depression. The performed analysis revealed that voluntary and involuntary singlehood was not predictive of any of the indicators of positive mental health and social dysfunction as an indicator of mental health illness.

In the second step, the mediator (romantic loneliness) was regressed on the predictor variable (voluntary and involuntary singlehood) to establish Path a (see Fig. 1). The performed analysis indicated that voluntary and involuntary singlehood was associated with romantic loneliness, explaining the 18 % of variance in romantic loneliness.

Finally, in the third step, in order to test whether romantic loneliness was related to each of the seven outcomes, the outcomes were regressed simultaneously on both romantic loneliness and the predictor (voluntary and involuntary singlehood). The coefficients associated with the associations between romantic loneliness and all seven outcomes (controlling for predictor) were nonsignificant. Thus, the condition for Step 3 was not met (Path b was nonsignificant). This third regression equation also provided an estimate of Path c’, the relation between predictor and seven outcomes, controlling for romantic loneliness. When that path is zero, there is complete mediation. However, Path c’ for all seven outcomes was not significant. Therefore, this final criterion in the mediation test was not met.

In sum, the analysis of mediation of romantic loneliness in the linkage between voluntary vs. involuntary singlehood, positive mental health, and mental health illness demonstrated that this mediation is not significant. There was significant direct relationship (Path c; see Fig. 1) between voluntary and involuntary singlehood and somatic symptoms (β = .16, *p* = .049), anxiety and insomnia (β = .21, *p* = .008), and severe depression (β = .18, *p* = .032), and significant direct relationship (Path a; see Fig. 1) between voluntary and involuntary singlehood and romantic loneliness (β = .43, *p* = .000).

Since the performed analyses revealed only direct effects of voluntary and involuntary singlehood on somatic symptoms, anxiety and insomnia, severe depression, and romantic loneliness, moderation analysis with gender as a moderator was performed only for those outcomes (see Table 5). The moderation analysis was performed in the PROCESS module within SPSS 23.

**Table 5:** Testing moderator effects of gender on the linkage between voluntary and involuntary singlehood and somatic symptoms, anxiety and insomnia, and severe depression

	*B*	*SE B*	*t*	*p*	*R* ^*2*^
Somatic symptoms					
Gender	−0.54	0.31	−1.71	.089	
Voluntary and involuntary singlehood	0.61	0.31	1.96	.051	
Gender x Voluntary and involuntary singlehood	−0.13	0.63	−0.21	.837	.00
Anxiety and insomnia					
Gender	−0.27	0.33	−0.83	.410	
Voluntary and involuntary singlehood	0.89	0.33	2.72	.007	
Gender x Voluntary and involuntary singlehood	0.57	0.66	0.09	.913	.00
Severe depression					
Gender	−0.23	0.26	−0.91	.366	
Voluntary and involuntary singlehood	0.53	0.26	2.09	.038	
Gender x Voluntary and involuntary singlehood	−0.06	0.52	−0.12	.907	.00

Results presented in Table 5 indicated that gender did not operate as a moderator of the linkage between somatic symptoms, anxiety and insomnia, and severe depression.

## Discussion

The objective of the present study was to investigate whether voluntary singlehood (singlehood by choice) and involuntary singlehood (singlehood not by choice) are related to romantic loneliness, positive mental health and mental illness in a group of single Polish young adults. In addition, special attention was paid to gender differences in regard to domain of romantic loneliness, positive mental health and mental health illness. The present study also intended to expand prior research on singlehood in young adulthood by exploring a theoretical model in which romantic loneliness was postulated to mediate between voluntary vs. involuntary singlehood and mental health outcomes.

The major findings obtained in the presented study did not provide evidence for the hypotheses (H1 and H2) predicting that voluntary single young adults will report higher level of positive mental health and lower level of mental health illness. The present study showed that voluntary and involuntary single young adults differed neither in regard to emotional, psychological, social well-being or total well-being, nor in regard to mental health illness (i.e., somatic symptoms, anxiety and insomnia, social dysfunction, severe depression, and total mental illness). At the same time, performed analyses supported the third hypothesis (H3) which assumes that voluntary single young adults experience lower level of romantic loneliness than involuntary single young adults. The results from the current study add to the complexity of singlehood captured from the perspective of its voluntary vs. involuntary nature. The lack of differences in the domain of positive mental health and mental health illness, contradict popular social stereotypes of singles perceived as miserable, unhappy, insecure, more neurotic, less satisfied with their lives, with lower self-esteem, less satisfied with their relationship status, and desiring to change their relationship status when compared to partnered individuals (DePaulo and Morris 2005; Greitemeyer 2009). This positive view of voluntary singlehood may be related to the fact that nowadays singlehood is often assumed to be an expression of individualization and individualistic attitudes and the expanded freedom of people’s choice (Poortman and Liefbroer 2010). Moreover, this new perception of singlehood as a consciously and voluntarily chosen lifestyle was noticed already by Stein in Stein 1975, who in his qualitative study analyzed singlehood as a positive choice made by adults who chose not to marry or re-marry. Furthermore, the results obtained in the present study seem to support observations made by some researchers that negative associations with singlehood may not be accurate, and that a more contemporary singlehood may represent choice and be associated with positive outcomes such as happiness (Keith 2003).

At the same time, the present study suggests that regardless of whether one’s singlehood is perceived as a result of personal choice or caused by some external circumstances, it is related to the experience of romantic loneliness; however, the level of this loneliness is lower among those who chose their singlehood. The association of romantic loneliness with voluntary vs. involuntary singlehood revealed in the current investigation may support the results from a prior study by Poortman and Liefbroer (2010), indicating that despite greater freedom that young adults nowadays have in the area of the possibility of shaping and directing their life paths, they generally choose to commit rather than to stay single (Poortman and Liefbroer 2010). Indeed, if young adults prefer being committed to remaining single, the experience of romantic loneliness by single individuals is not surprising. Furthermore, the presented results emphasize the significance of the need to belong as a fundamental human motivation (Baumeister and Leary 1995). Although this need can be satisfied in a variety of frequent positive interactions with other people within the context of long-term caring relationships (e.g., friendships, relationships with parents and siblings), during adulthood, romantic partners assume a special position in the network of attachment figures and become a primary attachment figure (Rowe and Carnelley 2005). Moreover, most people prefer to have a romantic partner than to be single (Greitemeyer 2009), and the vast majority of singles are more positive about living together than about living apart from a partner (Poortman and Liefbroer 2010). Therefore, considering that single individuals do not have a romantic partner, they experience romantic loneliness, especially when they do not perceive their singlehood as voluntary and depending on their personal decision.

In the current study two open research questions (RQ1 and RQ2) were formulated with respect to gender differences in the domain of positive mental health, mental health illness, romantic loneliness, and the possibility of interaction between voluntary and involuntary singlehood and gender. The performed analyses demonstrated no difference in the level of positive mental health or mental health illness, and no interactional effect of voluntary and involuntary singlehood and gender. Lack of gender differences in the domain of positive mental health and mental health illness may be related to contemporary changes of a diminishing pattern of gender differences in the sphere of intimacy during young adulthood (Feldman et al. 1998). These changes are thought to contribute to acknowledging the benefits deriving from intimacy and closeness with a partner by men (Feldman et al. 1998). Thus, as gender differences in the domain of romantic relationships appear to diminish, it is possible that men and women have similar experiences in the domain of romantic relationships, and as result, they experience similar levels of positive mental health and mental health illness. This explanation would be congruent with Simon and Barrett’ (2010) indication of the complexity of the association between non-marital romantic relationships and young adults’ mental health, which is of special importance in relation to the contemporary changes in young adults’ lifestyles, including being single, living apart together, and cohabitation without marriage (Lehnart et al. 2010), and in men’s and women’s roles (Simon 2002). These notions could also explain whether gender in the current study was not found to moderate the linkage between voluntary and involuntary singlehood, positive mental health and mental illness.

Although in the current study single women and men did not differ in the domain of positive mental health and mental health illness, they differed in regard to romantic loneliness which higher levels reported by women. In literature, women are depicted as having a stronger interest in establishing close, dyadic social ties (Feldman et al. 1998; Stokes and Levin 1986), and also, in line with commonplace beliefs, that men are less willing to connect with others than women (Schmitt 2008). As a result, single women, regardless of the nature of their singlehood (voluntary vs. involuntary), may experience higher romantic loneliness than single men.

Finally, in line with the fourth hypothesis, it was expected that romantic loneliness would mediate the linkage between voluntary and involuntary singlehood, positive mental health and mental health illness. The set of mediation analyses separately performed for emotional, psychological and social well-being (positive mental health) and for indicators of mental health illness (i.e., somatic symptoms, anxiety and insomnia, social dysfunction and severe depression) revealed that romantic loneliness does not operate as a mediator for the relationship between voluntary and involuntary singlehood, positive mental health and mental health illness. It is also possible that the mediating role of romantic loneliness was not detected in the current study due to the lower reliability of the scale used to measure romantic loneliness variable. As suggested in literature, the mediating variable should be measured with a reliability of at least .90. (Mallinckrodt et al. 2006). In result, lower reliability of the interaction term increases its standard error and reduces the power of the test (Frazier et al. 2004). Thus, the more reliable measure of romantic loneliness and larger sample sizes would have revealed mediation of romantic loneliness that was not discernible in the current study, but the present results suggest that such mediation may not exist.

The performed analyses demonstrated the direct relationships between voluntary and involuntary singlehood, somatic symptoms, anxiety and insomnia, severe depression, and romantic loneliness. Specifically, higher levels of these indicators of mental health illness (the weak associations) and higher level of romantic loneliness (the moderate association) were predicted by involuntary singlehood. Thus, the outcomes of involuntary singlehood do not seem to be so detrimental as Adelman and Ahuvia (1991, p. 273) pointed, writing that “Involuntary singleness can be a profound source of pain for many adults.” At the same time, the associations observed in the current study, however weak and moderate, suggest that when people have the need to possess a partner/spouse and when this need is unsatisfied, they may experience symptoms of psychological distress. For example, Mellor et al. (2008) found that the unmet need to belong was associated with loneliness, suggesting that a failure in satisfying belongingness needs may contribute to social isolation, alienation, and loneliness. Thus, in light of the obtained results, remaining single, if an individual’s choice, seems to be related to lower psychological distress.

### Limitation and Future Directions

Several factors specific to the present study limit the conclusions that can be drawn from this article. Several factors specific to the present study limit the conclusions that can be drawn. First, because of the cross-sectional nature of the study, it cannot be determined whether being single (voluntarily or involuntarily) is a cause or consequence of positive mental health and mental health illness, and longitudinal research is needed to evaluate the nature of these associations over time. This issue is of special importance in light of research indicating two possible explanations of the linkage between marital status and mental and physical health. Specifically, in line with *the social selection hypothesis*, better-adjusted, healthier people become and remain married, and this selection effect accounts for observed group differences between married and unmarried people (e.g., Horn et al. 2013). In particular, psychological well-being or mental health may influence the probability of staying in a marriage, and, in addition, less stable personality traits may enhance the risk of marital dissolution and contribute to lower psychological well-being (Mastekaasa 1994). In turn, in line with *the social causation hypothesis* marriage offers a variety of benefits which causes positive changes and/or protects against negative changes in mental or physical health (Horn et al. 2013). Thus, the lack of material resources is detrimental to the health among unmarried people (Wyke and Ford 1992). Prior longitudinal studies demonstrated that the above-mentioned hypotheses indicate that mental health is a consequence as well as a cause of marital status (e.g., Mastekaasa 1992). Regarding this issue, future research would benefit from longitudinal assessments of the relationship between status and mental health. Second, the sample size used in the current study is relatively small and consisted solely of heterosexual participants living in Poland. Therefore, the results may not generalize to individuals of other sexual orientations, in particular gay, lesbian, and bisexual young people whose mental health wellbeing may be at risk as suggested by Fergusson et al. (1999). This has also been suggested by a more recent study, in which among participants under the age of 35 years, lesbian/gay identity was associated with an increased risk of symptoms of common mental disorders (Semlyen et al. 2016). Third, Poland, despite many social changes regarding marital and family life, is still a country of traditional values, in which most adolescents and young adults desire to marry and have a successful marital and family life (e.g., Rostowski 2009). Therefore, this specific social and cultural context may impact the experiences associated with singlehood. Further research should include larger samples and participants from Western cultures to examine the possibility that the more individualistic and nontraditional social context has influence on the effects of voluntary versus involuntary singlehood. Forth, future studies, for instance in research of qualitative nature, should carefully consider subjective and objective reasons for singlehood, and their associations with loneliness and mental health. This issue may be of special concern regarding the diversity of single status and reasons for singlehood (i.e., never-married, divorced, separated, widowed) (Cotten 1999; DePaulo and Morris 2005), which may translate into various aspects of singlehood. For instance, in White’s study (Mastekaasa 1992), individuals who had always been single were in better health than people who were married, divorced, separated, or widowed. In addition, it is important to note that the distinction between personal choice and external circumstances as a cause of singlehood may not be so clear and evident, and it seems to be rather related to subjective perceptions of singlehood by an individual than merely to objective circumstances.

Despite these limitations and the initial nature of findings, the present study highlights the importance of further research on the voluntary and involuntary singlehood and young adults’ mental health and other correlates and outcomes such as self-esteem, perceived social support, attitudes towards one’s own singlehood and involuntary childlessness.
